# Exploring the utilization and perceptions of pre-travel health consultations in primary care settings in Saudi Arabia: a cross-sectional study

**DOI:** 10.1186/s40794-024-00223-2

**Published:** 2024-06-15

**Authors:** Naheel A. AlAmer, Amani M. AlQarni

**Affiliations:** Department of Family and Community Medicine, College of Medicine, Imam Abdulrahman Bin Faisal University, Dammam, Saudi Arabia

**Keywords:** Pre-travel health consultations, International travel, Travel health services, Saudi Arabia, Primary healthcare, Health risk assessment, Vaccination recommendations, Health education campaigns, Traveler perceptions

## Abstract

**Background:**

International travel exposes individuals to diverse health risks, necessitating proactive pre-travel health preparations. Saudi Arabia has witnessed increased outbound travel. This study addresses a critical gap in knowledge by investigating the utilization and perceptions of pre-travel health consultations among adults in the Eastern Province of Saudi Arabia.

**Methods:**

This cross-sectional study surveyed patients at the Family and Community Medicine Center of Imam Abdulrahman Bin Faisal University during January 2024 to explore perceptions of pre-travel health consultations among the Saudi Arabian population. Adults aged 18 years or older in the waiting area were invited to complete a self-administered questionnaire.

**Results:**

Of the 772 participants, 624 (80.8%) engaged in international travel within the last year. However, 593 (76.8%) had never sought pre-travel health consultations. Age, gender, and education level significantly influenced the pursuit of pre-travel health advice, with older individuals, females, and those with higher educational attainment more likely to seek consultations. Participants perceived vaccination recommendations (597, 77.4%) and disease prevention information (678, 87.8%) as crucial parts of pre-travel health consultations. However, barriers to seeking advice included perceived low risk (445, 74.8%), lack of awareness (215, 36.3%), time constraints (128, 21.6%), and cost concerns (92, 15.5%).

**Conclusion:**

The low prevalence of pre-travel health consultations among travelers highlights the need for targeted educational campaigns and the integration of travel health services into primary healthcare. Addressing the identified barriers and leveraging preferred information sources are crucial steps towards enhancing the uptake of pre-travel health consultations, ultimately improving the health and safety of international travelers from the region.

## Background

International travel has become increasingly prevalent in today’s globalized world, exposing individuals to diverse cultures, environments, and potential health risks. Saudi Arabia, as a hub for both business and leisure travel, has witnessed a surge in outbound international travel [1]. However, the complexities of health risks associated with international travel necessitate a proactive approach to pre-travel health preparations. Pre-travel health consultations serve as a crucial preventive measure, encompassing risk assessments, vaccinations, and health advice to mitigate potential health threats [2, 3].

The World Health Organization (WHO) underscores the significance of pre-travel health advice, emphasizing its role in preventing travel-related diseases and ensuring the well-being of individuals during and after their journeys [4]. Numerous infectious diseases, such as malaria, yellow fever, and typhoid, pose specific risks to travelers, particularly when journeying to regions with different epidemiological profiles [5–8].

As Saudi Arabia progresses under the Kingdom’s Vision 2030 and National Transformation Program, its healthcare system is undergoing significant reforms, with a particular emphasis on enhancing primary healthcare. This strategic shift aims to create a more effective, comprehensive, and integrated health system that prioritizes both individual and societal health [9]. Part of this reform focuses on improving primary health care centers, which are pivotal in delivering preventive health services, including those vital for international travelers. This enhancement is crucial given the demographic trends towards a younger, travel-inclined population and the nation’s role as a global business and cultural hub [10, 11].

Recognizing the global significance of pre-travel health consultations, it is essential to acknowledge studies conducted worldwide [12–16]. These studies have shed light on the practices and patterns of pre-travel health consultations. However, despite this wealth of international research, a critical research gap persists in understanding the nuanced practices within the unique context of Saudi Arabia. This study aims to fill this gap by conducting a comprehensive survey focused on the perceptions, barriers, and preferences related to pre-travel health consultations among the Saudi Arabian population.

The findings of this study have implications for public health policy, healthcare delivery, and travel medicine practice in Saudi Arabia. By identifying barriers and preferences related to pre-travel health consultations, the study may help inform the development of targeted interventions and educational campaigns, ultimately contributing toward the goal of a healthier and more informed traveling population.

## Methods

### Study design

This cross-sectional study analyzed data from all patients who attended the Family and Community Medicine Center of Imam Abdulrahman Bin Faisal University from January 1 to January 31, 2024. The study aimed to investigate the perceptions of pre-travel health consultations among the Saudi Arabian population, focusing on their awareness, attitudes, and behaviors regarding pre-travel health advice and services.

### Participant recruitment

Participants were approached in the waiting area of the Family and Community Medicine Center. This center serves as a primary care health center, offering a range of medical services to the community, and provides quality education and training in family medicine and community medicine for both undergraduate and postgraduate programs. The study targeted individuals aged 18 years or older, waiting for their scheduled appointments, to fill out a self-administered questionnaire. Prior to participation, individuals were briefed on the study’s objectives, the voluntary nature of their involvement, and the confidentiality of their responses. Informed consent was obtained from all participants. The questionnaire was made available in both English and Arabic to accommodate the linguistic preferences of the participants, ensuring inclusivity and comprehension. No compensation was provided to underscore the voluntary participation in the study.

### Survey content

The self-administered survey aimed to explore perceptions of pre-travel health practices among the Saudi Arabian population. The survey comprised five sections and was administered in paper format:

- The first section covered demographic and travel information, including age group, gender, education level, employment status, history of recent international travel within the last 12 months, and the number of international trips within the last 12 months.
- The second section addressed the utilization of pre-travel health consultations. It covered the frequency of seeking pre-travel consultations, factors influencing the decision to seek such consultations, and the perceived helpfulness of information received in previous pre-travel consultations.
- The third section focused on the perceived importance of pre-travel health consultations. Participants were asked to rate the importance of pre-travel health consultations using a 5-point Likert scale and were asked about the aspects of pre-travel health consultations that they find most valuable. Participants were also asked about their opinion on how the perceived importance of pre-travel health consultations could be increased among the general population.
- The fourth section explored the reasons for not seeking pre-travel health advice.
- The fifth section addressed preferences for receiving pre-travel health information. This included a question about sources of information preferred for pre-travel health advice and the format of information delivery preferred.

### Survey validation

Following expert reviews, a pilot test involving 30 individual’s representative of the study population was conducted to solicit feedback on clarity, relevance, and cultural appropriateness. The survey content was tailored to maintain a 50% similarity with a previous study to allow for comparative analysis. Changes were made to the survey based on pilot test insights, including clarifications and adjustments to enhance effectiveness.

### Sample size calculation

The sample size (*n* = 384) was calculated using the formula for estimating proportions, considering an estimated prevalence of seeking pre-travel health consultations (50%), a desired precision level (5%), a margin of error of 5%, and a confidence level of 95%.

### Ethical considerations

This study adhered to the ethical standards outlined in the Declaration of Helsinki. Participants were provided with detailed information about the study’s purpose, the voluntary nature of participation, and the confidentiality of their responses. Informed consent was obtained from all participants at the primary health centers before survey participation. Ethical approval was obtained from the Institutional Review Board at Imam Abdulrahman Bin Faisal University (Approval number: IRB-2024-01-040).

### Statistical analysis

Quantitative data collected from the survey were analyzed using IBM SPSS Statistics version 26. Descriptive statistics, including frequencies and percentages, were used to summarize the participants’ responses. Figures were utilized to provide a visual illustration of the responses. Additionally, multivariable logistic regression analysis was conducted to examine factors associated with the utilization of pre-travel health consultations, with results presented as odds ratios (OR) and 95% confidence intervals (CI). All statistical tests were two-sided, and a p-value of less than 0.05 was considered statistically significant.

## Results

### Demographic characteristics

The survey involved 772 participants, with a relatively even distribution of 420 (54.4%) men and 352 (45.6%) women. Concerning the age groups, 314 (40.7%) fell within the 31–45 years category, the most frequent age group, followed by 218 (28.2%) participants in the 18–30 years category. In terms of education, 422 (54.5%) participants had a college degree or were currently attending college, while 213 (27.6%) held postgraduate degrees. Regarding employment status, the majority, 513 (66.5%) participants, were employed.

Concerning international travel history, a significant proportion, 624 (80.8%) participants, engaged in international travel within the last 12 months. Specifically, 409 (53.0%) mentioned having 1–2 trips during the same period, while 140 (18.1%) participants reported 3–4 trips. The destinations of international trips reported by participants are summarized in Table 1.

**Table 1:** Demographic Characteristics of Study Participants (*N* = 772)

Category	Variables	*N*	(%)
Age Group (years)	18–30 years	218	(28.2%)
	31–45 years	314	(40.7%)
	46–60 years	139	(18.0%)
	61 or older	101	(13.1%)
Gender	Male	420	(54.4%)
	Female	352	(45.6%)
Education Level	High School or below	137	(17.7%)
	College/University	422	(54.7%)
	Postgraduate	213	(27.6%)
Employment Status	Employed	513	(66.5%)
	Unemployed	71	(9.2%)
	Student	188	(24.4%)
Number of International Trips (Last 12 Months)	None	148	(19.2%)
	1–2 times	409	(53.0%)
	3–4 times	140	(18.1%)
	5 or more times	75	(9.7%)
Destination of International Trips (Last 12 Months)	Middle East	321	(41.6%)
	Asia	175	(22.7%)
	Africa	89	(11.5%)
	Europe	201	(26.1%)
	Americas	60	(7.8%)

Abbreviations: N: number of participants

Note: Percentages may not add up to 100% due to rounding.

### Utilization of pre-travel health consultations

A considerable proportion of participants, 593 (76.8%), had never had any pre-travel health consultation, 37 (4.8%) had it on every international trip, while 87 (11.3%) sought such consultations occasionally. Among the 179 (23.2%) participants with previous pre-travel health consultations, 107 (59.8%) expressed satisfaction, finding the consultations helpful.

Participants highlighted diverse factors influencing their decision to seek pre-travel health consultations. Key influencers included recommendations from healthcare professionals, cited by 297 (38.5%) participants, previous travel experiences reported by 144 (18.7%) participants, and awareness of travel-related health risks by 178 (23.1%) participants (Table 2).

**Table 2:** Utilization and Perceptions of Pre-Travel Health Consultations (*N* = 772)

Category	Variables	*N*	(%)
Previous Pre-Travel Health Consultation	Yes	179	(23.2%)
	No	593	(76.8%)
Frequency of Seeking Consultations	Every Trip	37	(4.8%)
	Occasionally/Rarely	142	(18.4%)
	Never	593	(76.8%)
Factors Influencing Decision to Seek Pre-Travel Health Consultation	Recommendations From Healthcare Professionals	297	(38.5%)
	Awareness of Risks	178	(23.1%)
	Previous Experiences	144	(18.7%)
	Other/Not Specified	153	(19.8%)
Satisfaction with Consultation Information	Helpful	107	(59.8% of 179)
	Not Helpful	72	(40.2% of 179)

Abbreviations: N: number of participants

Note: Percentages may not add up to 100% due to rounding.

Multivariable logistic regression analysis was conducted to identify factors significantly associated with the likelihood of individuals seeking pre-travel health advice. This comprehensive analysis revealed that age significantly influences health-seeking behavior, with older age groups (46–60 years and 61 or older) showing a higher propensity to seek advice (OR = 2.2 [1.3–3.9], *p* < 0.01 and OR = 5.7 [2.9–14.2], *p* < 0.01, respectively) compared to the 18–30 years reference group. Gender differences were also pronounced; females were more likely than males to seek pre-travel health consultations (OR = 6.1 [4.1–9.7], *p* < 0.01). Education level emerged as another critical factor, especially among postgraduates, who were significantly more inclined to seek advice (OR = 14.5 [7.5–18.7], *p* < 0.01) compared to those with a high school education or below (Table 3).

**Table 3:** Factors associated with seeking of pre-travel health consultations

Category	Variables	Univariable Analysis	Multivariable Analysis	
		OR [95% CI]	OR [95% CI]	*P* value
Age Group (years)	18–30 years	Reference Group		
	31–45 years	0.8 [0.5–1.2]	0.7 [0.4–1.3]	0.14
	46–60 years	2.0 [1.2–3.3]	2.2 [1.3–3.9]	< 0.01
	61 or older	5.3 [3.1–8.9]	5.7 [2.9–14.2]	< 0.01
Gender	Male	Reference Group		
	Female	5.5 [3.8–8.1]	6.1 [4.1–9.7]	< 0.01
Education Level	High School or below	Reference Group		
	College/University	0.8 [0.4–1.4]	0.8 [0.3–2.5]	0.69
	Postgraduate	10.7 [6.0–19.4]	14.5 [7.5–18.7]	< 0.01
Employment Status	Employed	Reference Group		
	Unemployed	1.5 [0.9–2.6]	1.7 [0.7–2.9]	0.14
	Student	0.9 [0.6–1.4]	1.0 [0.5–1.2]	0.98
Recent International Travel	Yes	Reference Group		
	No	5.0 [3.4–7.3]	3.1 [0.9–6.7]	0.02
Number of International Trips	1–2 times	Reference Group		
	3–4 times	1.4 [0.9–2.0]	1.1 [0.7–2.9]	0.81
	5 or more times	0.8 [0.4–1.4]	0.7 [0.3–3.4]	0.58

Abbreviations: OR: odds ratio; CI: confidence intervals

### Perceived importance of pre-travel health consultations

The results revealed a spectrum of perspectives on the importance of pre-travel health consultations as assessed on a 5-point Likert scale. Specifically, 252 (32.6%) participants rated them as extremely important, 149 (19.3%) participants deemed them somewhat important, 62 (8.0%) participants expressed a neutral stance, 31 (4.0%) participants considered them slightly important, and 278 (36.0%) participants indicated that they considered pre-travel health consultations not important at all.

**Fig. 1:**
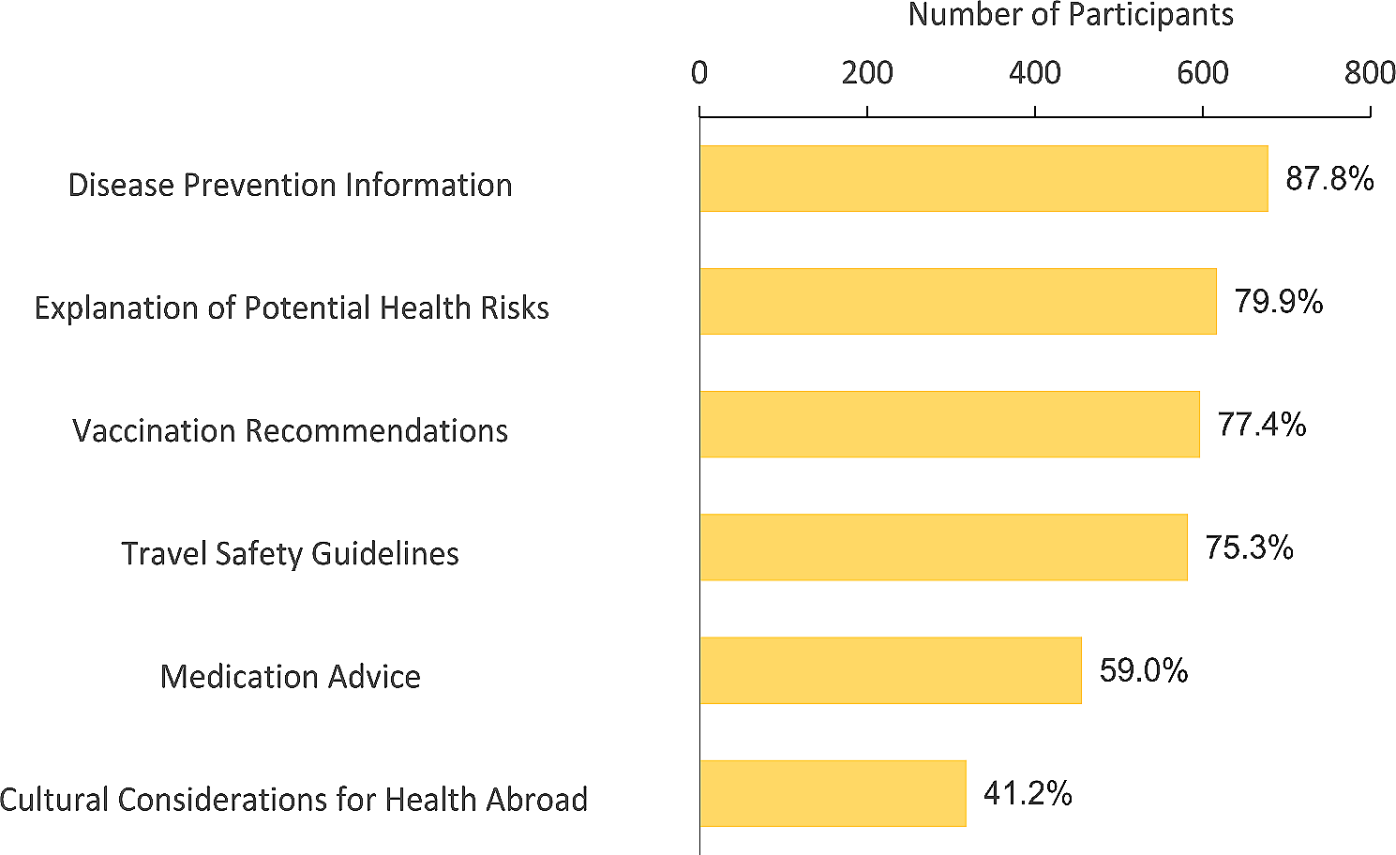
Perceived Importance of Aspects of Pre-Travel Health Consultations Among Study Participants (*N* = 772)

Participants were asked to indicate the perceived importance of various aspects within pre-travel health consultations. Figure 1 showcases the distribution of responses among participants. Disease prevention information emerged as the most notable focus, garnering attention from 678 (87.8%) participants. Travel safety guidelines closely followed, with 617 (79.9%) participants expressing interest. Vaccination recommendations were also highly regarded, with 597 (77.4%) participants. Explanations of potential health risks received substantial consideration from 582 (75.3%) participants. Medication advice was deemed important by 456 (59.0%) participants. Cultural considerations for health abroad were perceived as relevant by 318 (41.2%) participants.

The survey explored participants’ perspectives on strategies for enhancing the perceived importance of pre-travel health consultations within the general population. Notably, 620 (80.1%) participants advocated for increased awareness campaigns as a key approach. Collaboration with travel agencies also garnered significant support, with 450 (58.3%) participants endorsing this partnership. Accessible clinics and services were highlighted by 567 (73.4%) participants. Furthermore, 503 (65.1%) participants considered mandatory requirements for certain destinations essential. Regarding incentives, 382 (49.5%) participants suggested offering discounts or incentives for individuals seeking pre-travel health consultations. Engaging healthcare professionals in providing comprehensive and accessible pre-travel health information and services emerged as another impactful strategy, with 610 (78.9%) participants supporting this approach.

### Barriers to seeking pre-travel health advice

**Fig. 2:**
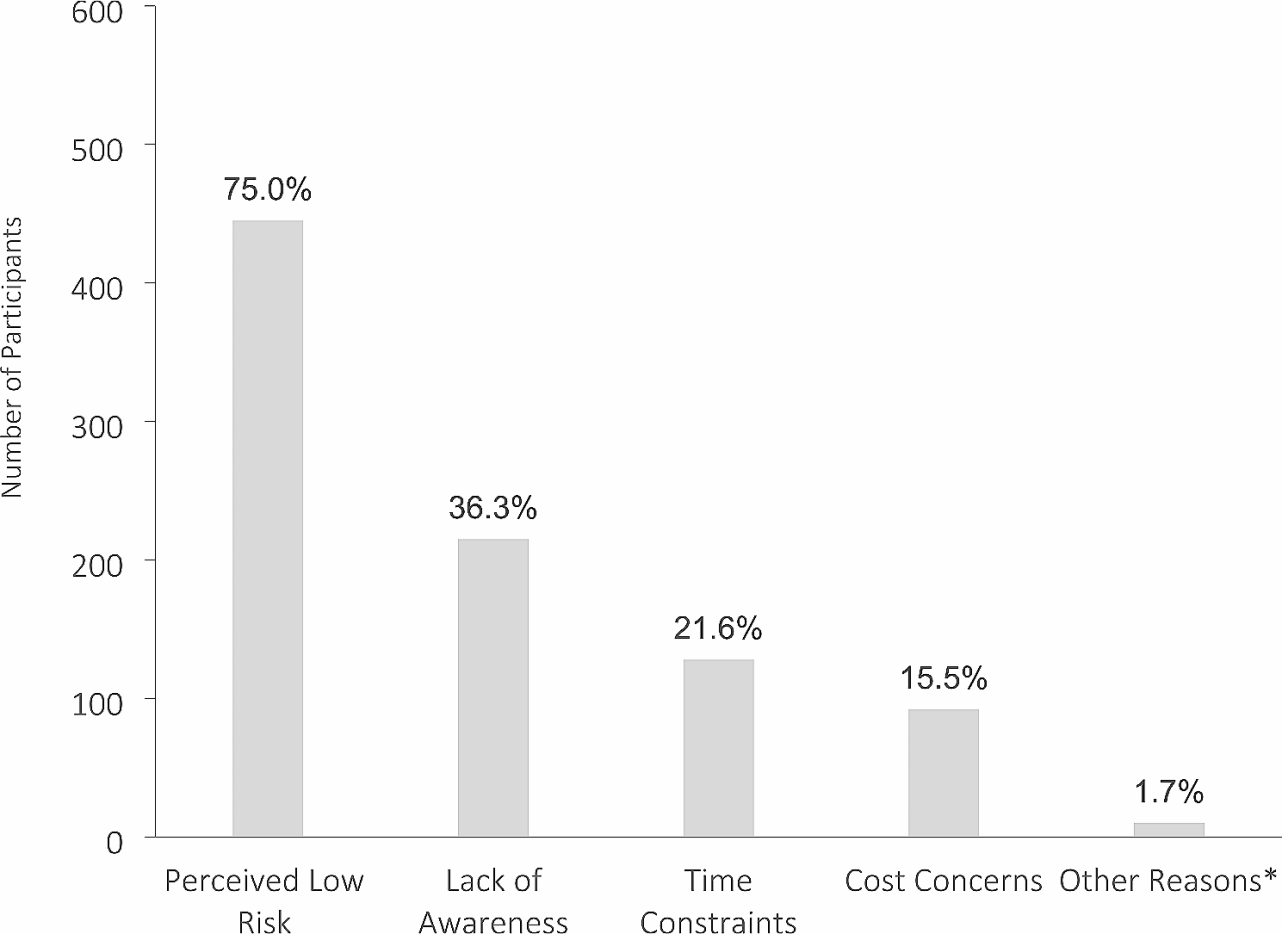
Barriers to Seeking Pre-Travel Health Consultation, Among Those Who Reported Never Attending a Pre-travel Health Consultation (*N* = 593). *Other reasons specified by participants who did not attend pre-travel clinic consultations: Personal beliefs (4 participants), reliance on alternative health practices (3 participants), and uncertainty about the effectiveness of pre-travel consultations (3 participant)

Among 593 participants who reported never attending pre-travel clinic consultations, the majority, encompassing 445 participants (75.0%), cited a perceived low risk as the primary reason. Other significant factors contributing to the decision included a lack of awareness (215 participants, 36.3%), time constraints (128 participants, 21.6%), and cost concerns (92 participants, 15.5%). A small proportion of participants (10 participants, 1.7%) specified other reasons (Fig. 2).

### Preferences for receiving pre-travel health information

In Table 4, participants’ responses regarding their preferred sources and formats for pre-travel health information are summarized. While online resources were indicated as the preferred source by a higher proportion of participants, with 688 (89.0%) participants expressing confidence in this medium, healthcare professionals were also highly favored, with 620 (80.1%) participants indicating trust in expert guidance. Additionally, travel clinics were considered valuable by 450 (58.3%) participants, while public health campaigns resonated with 315 (40.8%) participants.

**Table 4:** Participant preferences for receiving pre-travel health information (*N* = 772)

Category	Variable	*N*	(%)
Preferred Sources of Pre-Travel Health Advice	Online resources	688	(89.1%)
	Healthcare professionals	620	(80.3%)
	Travel clinics	450	(58.3%)
	Public health campaigns	315	(40.8%)
Preferred Formats of Information Delivery	Websites	612	(79.3%)
	Face-to-face consultations	425	(55.1%)
	Mobile apps	324	(42.0%)
	Brochures/handouts	178	(23.1%)

Abbreviations: N: number of participants

In terms of the format of information delivery, while face-to-face consultations were preferred by a substantial proportion of participants, with 425 (55.0%) participants opting for this personalized approach, other formats also garnered significant support. Brochures and handouts were preferred by 178 (23.1%), participants while websites proved to be a popular medium, capturing the preference of 612 (79.2%) participants. Additionally, mobile apps gained traction, with 324 (41.9%) participants expressing a preference for this convenient and accessible format.

## Discussion

The present study addresses a critical gap in the literature by providing insights into the perceptions, barriers, and preferences related to pre-travel health consultations among the Saudi Arabian population in the Eastern Province. The high prevalence of international travel, indicated by 80.8% of participants having traveled within the last 12 months, underscores the need for understanding pre-travel health practices in this population.

The relatively low prevalence of seeking pre-travel health consultations among participants, with 76.8% having never undergone such consultations, highlights a critical gap in the availability and accessibility of healthcare services tailored to the needs of travelers. This gap emphasizes the necessity for implementing more accessible and integrated healthcare solutions specifically designed to address the unique health concerns of individuals planning international trips. By recognizing and acknowledging this gap, healthcare providers can advocate for the integration of travel health services into primary healthcare settings, thereby ensuring that pre-travel health assessments and consultations are readily available to all individuals seeking comprehensive care [17]. Additionally, establishing dedicated travel health clinics or offering specialized services within existing healthcare facilities can enhance accessibility and encourage more individuals to seek pre-travel health advice. Ultimately, proactive measures to improve access to pre-travel healthcare services are essential for safeguarding the health and well-being of travelers and reducing the incidence of travel-related illnesses and complications.

The multivariable analysis model revealed that age and gender emerged as notable determinants of pre-travel health-seeking behavior. The increased propensity of older individuals to seek health advice may be attributed to greater awareness of health vulnerabilities and a higher perceived value of preventive measures with advancing age. This finding suggests that younger travelers might underestimate health risks associated with travel or may feel invulnerable to travel-related health issues, highlighting a crucial area for targeted health education and intervention. Furthermore, the pronounced likelihood of females seeking pre-travel health consultations compared to males could reflect gender differences in health awareness and risk perception. Education level, particularly among postgraduates, significantly influenced the pursuit of pre-travel health advice, suggesting that higher educational attainment may correlate with a better recognition of the risks associated with international travel and the benefits of preventive health consultations. This insight points to the potential of educational interventions in enhancing the uptake of pre-travel health services, especially among populations with lower educational backgrounds [18].

Participants demonstrated varying perspectives on the importance of pre-travel health consultations, with a substantial number considering them extremely important. The emphasis on vaccination recommendations, disease prevention information, medication advice, travel safety guidelines, and explanations of potential health risks reflects the multifaceted nature of health concerns among travelers. However, the primary barrier to seeking pre-travel health consultations identified in this study was the perceived low risk, as reported by a significant proportion of participants.

The perception of low risk for infections may indicate a lack of awareness regarding the potential health threats associated with international travel. This finding is consistent with previous studies. For example, a survey by AlOwaini et al. [19] on Saudi travelers to malaria-endemic countries revealed suboptimal knowledge among participants (42.7%), with a concerning finding that only 11.3% sought pre-travel health advice on malaria. Similarly, Kain et al. [20] conducted a systematic review on pre-travel health-seeking behavior and adherence to advice, revealing that the main hindrance is travelers’ perceived low risk of infection. Heywood et al. [21] observed that Asian travelers were less likely to seek pre-travel health advice and uptake pre-travel vaccines compared to Australian or other Western travelers. This discrepancy underscores the influence of regional factors, such as cultural norms, access to healthcare services, and awareness of travel-related health risks, on individuals’ perceptions and behaviors regarding pre-travel health preparation.

While our study investigated the perceptions, barriers, and preferences related to pre-travel health consultations among the Saudi Arabian population, it is important to build on previous studies conducted on the knowledge and practice of travel medicine among primary healthcare physicians in Saudi Arabia. For example, a study conducted by Sharahili et al. [22] revealed suboptimal knowledge and practice among healthcare professionals in Riyadh on travel medicine. Furthermore, Alduraibi et al. [23] focused on addressing the challenges faced by individuals with type 2 diabetes during travel. Their study revealed significant knowledge and practice gaps among primary healthcare physicians regarding how to best advise individuals with underlying health conditions on protecting against or avoiding travel-related health risks. This underscores the need for targeted education and training for healthcare professionals in Saudi Arabia to ensure comprehensive care for travelers with specific health concerns.

Understanding the preferred sources and formats for receiving pre-travel health information provides valuable insights for tailoring educational campaigns. The overwhelming preference for healthcare professionals and online resources indicates the importance of leveraging these channels for disseminating information. Collaboration with travel agencies and the promotion of accessible clinics align with the preferences of a substantial portion of the population, suggesting potential avenues for intervention.

While the study provides valuable insights, several additional limitations should be acknowledged. Firstly, the survey was conducted in only one center, and data collection occurred over a relatively short period of one month, potentially limiting the generalizability of the findings. Future research could address these limitations by conducting multi-center studies over longer durations. Furthermore, exploring the knowledge of travel medicine among primary care physicians in relation to the burden of disease among the population could provide valuable insights for targeted interventions and healthcare planning.

## Conclusion

In conclusion, our study highlights a significant gap in the uptake of pre-travel health consultations among the Saudi Arabian population in the Eastern Province, despite the high prevalence of international travel. The findings underscore the need to enhance awareness and accessibility of pre-travel health services. Strategies such as integrating travel health into primary healthcare should be considered to address these gaps. Age, gender, and educational level emerge as key determinants influencing the likelihood of seeking pre-travel health advice, indicating targeted interventions are necessary to address these disparities. By emphasizing the role of family doctors and primary care providers in promoting pre-travel health assessments as part of comprehensive care, we can pave the way for improved preventive health behaviors among travelers. Future efforts should focus on leveraging educational campaigns and healthcare provider engagement to mitigate perceived barriers and increase the perceived value of pre-travel health consultations. This study not only sheds light on the current practices and perceptions towards pre-travel health in Saudi Arabia but also sets the stage for future research and policy-making aimed at safeguarding the health of international travelers from the region.

## Data Availability

The datasets used and/or analyzed during the current study are available from the corresponding author upon reasonable request.
